# Obstacles to Enforcing Regulations Requiring the Tuberculin Test in Inter-State Cattle Traffic1Read at the Thirty-seventh Annual Meeting of the American Veterinary Medical Association, Detroit, September, 1900.

**Published:** 1900-10

**Authors:** Austin Peters

**Affiliations:** Chairman of the Massachusetts Cattle Commission


### OBSTACLES TO ENFORCING REGULATIONS REQUIRING THE TUBERCULIN TEST IN INTER-STATE CATTLE TRAFFIC.[1]

By Austin Peters, M.R.C.V.S.,
CHAIRMAN OF THE MASSACHUSETTS CATTLE COMMISSION.

Massachusetts was among the first States, if not the first State, requiring cattle brought within her borders to be kept for dairy or breeding purposes to be subjected to the tuberculin test, although for several years prior to the use of tuberculin as a diagnostic agent Maine had maintained a quarantine against all Massachusetts cattle because of the prevalence of tuberculosis in the old Bay State.

In 1894 the Massachusetts Legislature passed an act providing that owners should be reimbursed by the State for one-half the value of cattle killed by order of the Cattle Commission as having tuberculosis. In 1895 the law was amended so as to provide that owners should be paid full appraised value for tuberculous cattle

[1] Read at the Thirty-seventh Annual Meeting of the American Veterinary Medical Association, Detroit, September, 1900.

up to a limit not exceeding sixty dollars for any one animal. In 1899 this limit was reduced to forty dollars, the appraisal to be based upon the actual market value of the animal for milk or beef purposes at the time of condemnation, breeding not being considered. No compensation, however, is allowed for a diseased animal that has not been owned continuously within the State for six months prior to the time of condemnation.

It was during 1894, also, that the Cattle Commission commenced using tuberculin on a large scale as a diagnostic agent, killing all reacting animals. It was at once obvious that if the State was to undertake the extirpation of bovine tuberculosis only healthy animals should be brought into the Commonwealth to replace those killed, and that their condition of health must be based upon their standing the tuberculin test. Massachusetts does not raise a great deal of neat stock; the supply of milch cows is brought in largely from without the State, especially at the eastern end, where the milk producers depend almost entirely upon new purchases brought in from other States to keep up their dairy stock. These cows come largely from Maine, New Hampshire, and Vermont, quite a number come from New York State, and a few from other places.

Every Wednesday a large cattle market is held at Brighton, a suburb of Boston, at which there are often 700 or 800 cows. Of these 200 to 250 come from Maine, 100 to 125 from New Hampshire, as many more from Vermont, and a carload or two from New York State; these are practically all new milch cows. The rest come from Massachusetts, many of them brought in by milkmen to sell because they are farrow, gargetty, or otherwise worn out, most of them being sold for cheap beef or bolognas, their owners replacing them with fresh stock mainly from the northern New England States.

There are about 20,000 head of cattle from without the State (not counting beeves), mainly milch cows, passing through Brighton market each year; most of them remain in Massachusetts, quite a number go to Rhode Island, and a few are taken to Connecticut. The Cattle Commission, therefore, in the autumn of 1894, issued regulations requiring all persons bringing cattle into Massachusetts to have a permit unless brought to the stockyards at Brighton, Watertown, or Somerville, which were designated by the board as quarantine stations, and requiring all cattle, except beeves for immediate slaughter and calves under six months old, to be subjected to the tuberculin test.

Commencing November 21, 1894, the cattle arriving at the stockyards were held in quarantine and tested by the Commission, all reacting animals being killed. Of course, under the law there is no compensation for a tuberculous animal that has not been owned in the State for six months; but if an animal killed by order of the Commission is found free from disease the State has to pay its full value to the owner.

Under the method first adopted it was found that quite a number of animals gave an apparent reaction to tuberculin, which when killed showed no lesions of disease, and, therefore, had to be paid for, making the work quite expensive for the State. This was due to the fact that many cows, as the result of the excitement of transportation and strange surroundings, would have a rise of temperature the day after arriving that could easily be mistaken for the rise of a tuberculin reaction. The cattle trains arrive early Tuesday morning; the cows are unloaded and given twenty-four hours to rest and bag up, and are placed on the market Wednesday. Wednesday has been market day at Brighton from time immemorial, I was going to say; at least, it probably has been ever since there was a market at Brighton. In order to give the cattle time to rest and recover from the effects of transportation the Cattle Commission had market day changed to Thursday, the cattle being tested Tuesday evening and temperatures taken Wednesday; even this was not satisfactory.

It was then proposed that the cattle should be brought down a week ahead—that is, cattle intended for sale one week should be brought down the preceding week and held in quarantine six days, and then tested. This plan would have entailed an extra expense that the drovers could not have stood, as it would have upset their plans and cut into their profits to an extent that would have driven them out of business. After testing the cattle at Brighton from November 21, 1894, to April 30, 1895, with the drovers fighting, objecting, and placing every obstacle in the path of the Cattle Commission that they possibly could, the work was temporarily abandoned. In July, 1895, it was decided that milch cows and breeding stock coming into Massachusetts must be tested, but that each drover could employ a veterinarian to test the cattle before shipment, the examiner to make out a certificate of tuberculin test on blanks furnished by the Cattle Commission. These blanks are made in duplicate, the animal described therein is identified and released by a member of the Commission at the stockyards, who gives the owner the original and keeps the duplicate to file away where it can be referred to at any time if a question concerning a particular cow arises. At the present time each cow is required to have an ear-tag (furnished the drovers at cost by the Commission), the ear-tag number and certificate number having to correspond; this makes the identification of each animal more easy.

The drovers entered readily into this plan, and each arranged to have a veterinarian in his locality test his cattle. The Cattle Commission obtained a list of veterinarians from the commissioners of the other States, whom they considered reliable, and the intention at first was to have only veterinary graduates upon it, and only those vouched for by Cattle Commissions of their respective States. In some localities there were no qualified veterinarians, and it was arranged to accept tests of members of the laity who were practical cattlemen, castrators, and the like, and familiarized themselves with the proper methods of applying tuberculin. This work was done honestly, probably, for a few months, then crooked work commenced and has been carried on to a greater or less extent by some men ever since. (An honest quack is better than a dishonest graduate.)

This plan has been followed now for five years, the animals brought to the stockyards each week need no permit; the cow dealers give the certificates of tuberculin test (often fake ones) to the Commissioner having charge of this branch of the work, who identifies and releases the animals. Cattle brought to any other points can come in only on permits, and if over six months old and for dairy or breeding purposes must be tested either before shipment, or after arrival at their destination, at the expense and risk of the owner. If any cows are brought to the stockyard quarantine stations untested they are held and tested in five or six days, in time to go on to the market the next week. Any that react are killed; if slightly diseased the owner can have what the butcher will allow him for the beef; if badly diseased the carcass is tanked. If the Commission makes a mistake by killing a healthy animal it pays for it.

Since 1894 and 1895 many other States have adopted regulations based upon those of the Massachusetts Cattle Commission. The Bureau of Animal Industry of the United States Department of Agriculture requires all cattle held at the Government quarantine stations to be tested with tuberculin if over six months old. The Canadian Government also requires neat cattle brought into Canada to have a certificate of tuberculin test made by a government veterinarian in the country from which they are shipped; in the absence of this they are held and tested at the quarantine station at the port of entry.

One would suppose from this that the State of Massachusetts had a right to adopt such rules and regulations as were deemed necessary for the protection of her live-stock interests, yet the Commission has had a steady fight on its hands for the last six years with the cattle dealers and drovers.

The regulations regarding the cattle traffic in various States differ somewhat. In Massachusetts the law gives the Cattle Commission power to issue all necessary rules and regulations for the protection of the live-stock interests of the State; the same is true of Vermont, New Hampshire, and Colorado. In some of the other States the Governor issues a proclamation upon the recommendation of the live-stock sanitary boards; Illinois, Texas, Wisconsin, and several other States are examples of this method. In Maine the Board of Cattle Commissioners may issue the necessary rules and regulations, subject to the approval of the Governor.

In some States the importation of cattle is regulated by the public statutes; examples of this are Rhode Island, Connecticut, New Jersey, and Pennsylvania. This legislation may favor the tuberculin test or may be directly opposed to it, and may even be carried so far as to show a distinct animus against the veterinary profession. The State of Connecticut is the most striking example of this feeling, its law being an epitome of prejudice and ignorance. It is a result of the reaction that set in in 1896 against the tuberculosis scare, and provides for the appointment of a single cattle commissioner, and specifically stipulates that he shall be a farmer and stock-raiser of at least ten years' practical experience. Under the law he has the power to employ such assistants as he may need, but with the present prejudice it is not likely that many of them will be veterinarians. He is to be notified by any one bringing cattle into the State of the number and physical condition of such animals within six days of their arrival. (It is not likely that many people trouble themselves to do so.) Under her law Connecticut deserves to be the dumping-ground for tuberculous cattle from the other New England States, and it is not unlikely that in time this may be the case.

Rhode Island has an intelligent and conscientious Cattle Commission, the Secretary of Agriculture acting as its secretary, with a commissioner from each of the six counties, an appraiser, and a consulting veterinarian. Until this year the law of Rhode Island provided as follows :

CHAPTER 342, ACTS OF 1896.

SEC. 2. All persons, corporations or companies intending to ship, transport or drive cattle into the State, must produce a certificate to the effect that the cattle to be so shipped, transported or driven are free from tuberculosis as far as may be determined by physical examination and the tuberculin-test. The certificate shall give a description of each animal brought into the State, sufficiently accurate for identification, and shall also give the date and place of examination, the preparation of tuberculin used, the quantity injected, the temperature immediately before inoculation, the temperature at the eleventh hour, and every two hours subsequent thereto, for at least ten hours, or until the reaction is completed. The certificate shall be signed by a veterinarian who is a graduate of a recognized veterinary college, and shall be sent immediately to the Secretary of the State Board of Agriculture, who shall immediately notify a commissioner of the county into which the cattle are to be shipped, transported, or driven, and said commissioner shall examine the cattle to identify them. Failure to comply with the law shall be considered a misdemeanor punishable by a fine not to exceed one hundred dollars.

SEC. 3. Complaint for the violations of the provisions of this chapter shall be made to the Secretary of the State Board of Agriculture, and said Secretary shall be exempt from giving surety for costs on any complaint made as aforesaid.

From an intelligent stand-point this would seem to be a good law, and one which ought to have been left alone; but the Rhode Island Legislature of 1900 passed the following amendment:

CHAPTER 756, ACTS OF 1900.

SEC. 1. All persons desiring to import cattle into this State or from other States without obtaining the certificate required by Section 2 of Chapter 344 of the Public Laws, shall give written notice to the cattle commissioner of the county into which the cattle are brought within forty-eight hours after the arrival into the State of such cattle, and such notification shall contain a specified list of the cattle so imported, with a full description of age, sex, and such other particulars as may be necessary for the identification of the said cattle, and the place where they can be found.

SEC. 2. Immediately upon the receipt of such notification the cattle commissioner of the county into which said cattle are imported shall proceed within seventy-two hours to the place designated and make a physical examination of said cattle; and if upon such examination said cattle shall be deemed free from tuberculosis it shall be so certified by said cattle commissioner upon a permit, and a duplicate thereof to be given to the owner of said cattle, and the cattle shall be released for the use and benefit of the owner.

SEC. 3. If after such examination the cattle commissioner shall be of the opinion that the cattle so examined are afflicted with tuberculosis, he shall require of the importer that the suspected cattle be tested with tuberculin, said test to be applied by a veterinarian of a recognized veterinary college, who shall give to the said commissioner a certificate in writing that such test has been applied, together with a statement of the tuberculin used, quantity injected, temperature of each animal before inoculation, and at the eleventh and every two subsequent hours thereafter, for at least ten hours, or until reaction is complete; and a duplicate thereof shall be given to the owner of said cattle, and the original certificate shall be sent by the said commissioner to the Secretary of the State Board of Agriculture. If after such test it shall be proved that such suspected cattle are afflicted with tuberculosis, such diseased cattle shall be immediately slaughtered, upon written order of said commissioner, and the State shall not be required to compensate the owner for their loss, and the owner shall pay for testing such cattle with tuberculin; but if such cattle shall be found free from tuberculosis they shall be released for the use and benefit of the owner. If any of such cattle are slaughtered, and upon post-mortem examination it shall be found that the slaughtered animal was not afflicted with tuberculosis, then the animal so killed shall be paid for by the State at the full appraised value, in accordance with the provisions of Section 11 of Chapter 99 of the General Laws.

SEC. 4. Any person violating any of the provisions of this shall be deemed guilty of a misdemeanor, and shall be fined not more than one hundred dollars.

SEC. 5. This act shall take effect from and after its passage.

It can be readily seen that this law is intended to counteract that of 1896, and was passed in the face of the opposition of the Rhode Island Cattle Commission and all intelligent argument that could be brought to bear against it. This is another example of obstacles to the tuberculin test on the part of the cattlemen, and an instance, which is becoming too frequent in our legislative bodies, of a few men whose financial interests are interfered with being able to secure legislation that is opposed to the public wellbeing.

On the other hand, Pennsylvania and New Jersey have very good statutes for the protection of their live-stock interests, providing that all persons and corporations must have permits to bring cattle within their limits, and that cattle for dairy and breeding purposes must be tested with tuberculin, either before shipment by reliable veterinarians or else be held in quarantine and tested after arrival at their destination.

These laws will probably stand, provided the financial and corporate interests antagonized by them are not sufficiently powerful to attempt their modification or repeal. Probably legislation such as has been enacted in Pennsylvania and New Jersey is more efficacious for the protection of the live-stock interests of a State than the power to make rules and regulations given to cattle commissions or live-stock sanitary boards, because, first, there is more respect for statute laws than for the rules and regulations of a commission; and, secondly, the courts will take more interest in enforcing the law than they will in imposing penalties for breaking rules and regulations formulated by a commission.

(To be continued.)

